# High expression level of T-box transcription factor 5 predicts unfavorable survival in stage I and II gastric adenocarcinoma

**DOI:** 10.3892/ol.2015.3515

**Published:** 2015-07-20

**Authors:** YAN ZHENG, YUAN-FANG LI, WEI WANG, YONG-MING CHEN, DAN-DAN WANG, JING-JING ZHAO, QIU-ZHONG PAN, SHAN-SHAN JIANG, XIAO-FEI ZHANG, SHU-QIANG YUAN, HAI-BO QIU, CHUN-YU HUANG, BAI-WEI ZHAO, ZHI-WEI ZHOU, JIAN-CHUAN XIA

**Affiliations:** 1 Sun Yat-sen University Cancer Center, Guangzhou, Guangdong 510060, P.R. China; 2 Department of Thoracic Surgery, Henan Cancer Hospital, The Affiliated Cancer Hospital of Zhengzhou University, Zhengzhou, Henan 450008, P.R. China; 3 National Laboratory for Bio-Drugs of Ministry of Health, Provincial Laboratory for Modern Medicine and Technology of Shandong, Research Center for Medicinal Biotechnology, Shandong Academy of Medical Sciences, Jinan, Shandong 250062, P.R. China

**Keywords:** T-box transcription factor 5, gastric adenocarcinoma, prognosis factor

## Abstract

The expression of T-box transcription factor 5 (TBX5) has previously been observed in human cancer. The aim of the present study was to investigate TBX5 expression and its potential clinical significance in gastric cancer (GC). Using reverse transcription-quantitative polymerase chain reaction, the TBX5 mRNA expression levels in 30 pairs of surgically resected healthy gastric tissues and early stage (stages I and II) GC tissues were evaluated. The TBX5 mRNA expression levels were increased in GC stage I and II tumor tissues (P=0.01, n=30) compared with the matched adjacent non-tumor tissue. However, no significant difference was observed in TBX5 mRNA expression levels in matched adjacent non-tumor tissue compared with the tumor tissue from stage III and IV GC samples (P=0.318, n=30). Immunohistochemical analysis for TBX5 expression was performed on 161 paraffin-embedded stage I and II GC tissue blocks. Statistical analysis was performed to evaluate the associations between TBX5 expression, clinicopathological factors and prognosis. Patients with stage I and II GC and tumors with high TBX5 expression levels presented poor overall survival (OS) rate (P=0.024). The Cox proportional hazards model analysis demonstrated that TBX5 expression was an independent risk factor (P=0.017). The present study indicates that high expression of TBX5 is associated with unfavorable OS rates in patients with stage I and II GC. In conclusion, the expression of TBX5 may be a valuable biomarker for the selection of cases of high-risk stage I and II GC.

## Introduction

Gastric carcinoma (GC) is a common type of cancer with an increasing incidence of malignancy in developing countries. More cases are diagnosed in China each year compared with other countries (1), and it is the second most common type of cancer-associated mortality in China at present (2). Different survival rates of patients with the same tumor node metastasis (TNM) scores have been observed in clinical observation. Therefore, the staging system of the American Joint Committee on Cancer may not be sufficient to predict clinical outcomes, as it does not consistently distinguish which patients may have a poor prognosis within the same stage. Increasing numbers of biomarkers have been reported that are associated with different types of cancer (3). The discovery and understanding of tumor-associated biomarkers may aid in improving the diagnosis of GC and the efficacy of treatments. In addition, this information may be used to select the most appropriate therapy, which is particularly important for patients with early-stage GC.

T-box transcription factors (TBXs) are a conserved gene family that are required for the embryonic development of the heart and forelimbs. TBX5 is critical for forelimb development and cardiogenesis (4,5) and is associated with Holt-Oram syndrome (HOS) (6,7). Yu *et al* (8) reported that TBX5 may be a potential tumor suppressor gene in colon cancer. However, Rosenbluh *et al* (9) indicated that a β-catenin/yes-associated protein 1 (YAP1)/TBX5 complex was required for the survival of cancer cells, particularly for the initiation and progression of colon cancer. However, the prognostic, clinical and pathological significance of TBX5 in human GC has not yet been identified. In the present study, the mRNA level of TBX5 was evaluated by reverse transcription-quantitative polymerase chain reaction (RT-qPCR) in 60 pairs of surgically resected GC and healthy gastric tissues. Data from a large cohort of patients with GC were used to evaluate the prognostic and clinicopathological value of TBX5 expression by immunohistochemistry.

## Materials and methods

### 

#### Ethics statement

The study was officially approved by the Ethics Committee of Sun Yat-sen University Cancer Center (Guangdong, China). Written informed consent from the patients/patient's families were obtained.

#### Patients

A total of 161 consecutive patients with histologically diagnosed stage I and II GC that underwent surgery between January 2003 and December 2006 were retrospectively evaluated and the paraffin-embedded samples were obtained. A total of 60 self-pairs fresh frozen tissue samples were obtained between June 2011 and January 2012 from the tumor tissue bank for reverse transcription-quantitative polymerase chain reaction (RT-qPCR) analysis. Patients who possessed a second primary tumor, previous malignant disease, died of postoperative complications or received neoadjuvant/adjuvant treatments, were excluded. The surgical procedures were performed by experienced surgeons using the Japanese Gastric Cancer Association guidelines (10).

#### Tissue specimens

A total of 30 self-pairs each of early-stage (stage I and II; collected between June 2011 and January 2012) and a total of 30 self-pairs each of late-stage (stage III and IV; collected between October 2011 and April 2012) gastric adenocarcinoma specimens and adjacent non-cancerous tissues were snap-frozen and stored at −80°C following surgery. The paraffin-embedded samples for immunohistochemisrty (IHC) were obtained from a total of 161 consecutive patients with histologically diagnosed stage I and II GC that underwent surgery between January 2003 and December 2006. The patients were previously untreated with no distant metastasis and had histologically proven GC of different stages.

#### Extraction of total RNA and RT-qPCR

The total RNA was extracted using TRIzol solution (Invitrogen Life Technologies, Carlsbad, CA, USA) according to the manufacturer's instructions. DNA contamination was eliminated by using RNAse-free DNAase. RT-qPCR was performed using the Maxima First Strand cDNA Synthesis Kit for RT-qPCR (Thermo Fisher Scientific, Inc., Waltham, MA, USA). For the reverse transcription (RT) reaction, 2 µg total RNA was used to synthesize first strand cDNA. After that the cDNA was used as template for RT-qPCR detection, which was performed using the SYBR Green PCR Master Mix (Invitrogen Life Technologies, Carlsbad, CA, USA). For the evaluation of the association between the GAPDH (internal control) and TBX5, the primer sequences were as follows: TBX5, F 5′-TCCACCCAACCCATACCC-3′ and R 5′-GCTGTGCCGACTCTGTCCTGT-3′; GAPDH, F 5′-CTCCTCCTGTTCGACAGTCAGC-3′ and R 5′-CCCAATACGACCAAATCCGTT-3′. A RT-qPCR machine (ABI 7900HT; Applied Biosystems Life Technologies, Foster City, CA, USA) that measured the binding of SYBR Green I to double-stranded DNA was used to perform gene-specific amplification. The cycling conditions were as follows: initial step at 95°C for 10 min, then 45 cycles of 95°C for 30 sec and at last 60°C for 60 sec. The instrument's software (SDS 2.0; Applied Biosystems Life Technologies) was used to calculate the amplicated sample's relative quantity.

#### Immunohistochemistry

Paraffin-embedded sections (2-µm thick) were put into the graded ethanol washes (through 100, 95, 90, 80 and 70% ethanol) to deparaffinize and rehydrate the samples. Antigen retrieval was then performed as follows: The slides were boiled in EDTA (1 mM; pH 8.0) for 15 min in a microwave oven. The sections were placed into 0.3% hydrogen peroxide solution for 10 min at room temperature. Next, the sections were washed with PBS and incubated overnight at 4°C with a 1:600 dilution of rabbit anti-human TBX5 polyclonal IgG antibody (LifeSpan Biosciences, Inc., Seattle, WA, USA). Following 3 washes with PBS, the secondary antibody was applied for 30 min at room temperature. Subsequently, the slides were developed with 3-diaminobenzidine tetrahydrochloride (Tianjin Fuyu Fine Chemical Co., Ltd., Tianjin, China). The sections were counterstained with 20% hematoxylin (Shanghai Huntz Enterprises, Inc., Shanghai, China) and then the slides were dehydrated and cleared.

#### Semi-quantitative methods

For immunohistochemical analysis, TBX5 expression was evaluated according to the percentage of positively stained cells. The scores of staining intensity were defined as ‘3’ (strongly stained; strikingly positive at low magnification); ‘2’ (moderately stained; visible at low magnification); ‘1’ (weakly stained; visible at high magnification); or ‘0’ (no staining). The positive percentage score was as follows: ‘3’ (>50%, diffuse); ‘2’ (25–50%, focal); ‘1’ (5–25%, sporadic); or ‘0’ (<5%, negative). Positive percentage score × staining intensity score = total TBX5 score. A total score of ≥4 was defined as high expression and <4 as low expression. Three investigators (Dr Yan Zheng, Dr Dan-Dan Wang and Dr Wei Wang) who were blind to the clinical outcomes independently evaluated TBX5 staining under a light microscope (Nikon Ecli, PSE 80i; Nikon Corporation, Tokyo, Japan). The results between the observers differed in ≤15% of the examined slides.

#### Follow-up

The surveillance studies following pulmonary resection included clinical and laboratory examinations every 3 months for the first 2 years, every 6 months for the next 2 years, and every 12 months thereafter until the patients were lost in follow-up (the patient could not be contacted) or patient mortality. The overall survival (OS) was used as a measure of prognosis, which was defined as the time from the surgery to mortality or the final follow-up.

#### Statistical analysis

All statistical analyses were performed with SPSS software, version 17.0 for Windows (SPSS Inc., Chicago, IL, USA). A Wilcoxon matched- pairs signed-rank test was used to compare the TBX5 protein levels in the tumor tissue and the adjacent normal tissue samples. The correlation between TBX5 and the clinicopathological characteristics were assessed using the χ^2^ test. Survival curves were plotted by the Kaplan-Meier method with the log-rank test. P≤0.05 was considered to indicate a statistically significant difference.

## Results

### 

#### RT-qPCR analysis

**Figure 1.**
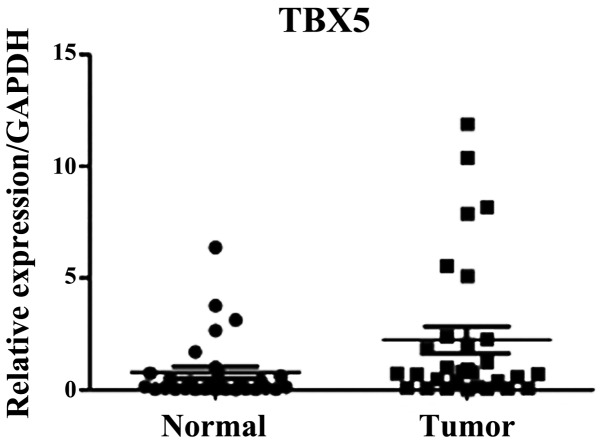
Increased TBX5 mRNA expression in stage I and II gastric cancer tumor tissues was detected by reverse transcription-quantitative polymerase chain reaction in tumor vs. adjacent non-tumor tissues (P<0.05, Wilcoxon matched-pairs signed-rank test). TBX5, T-box transcription factor 5.

**Figure 2.**
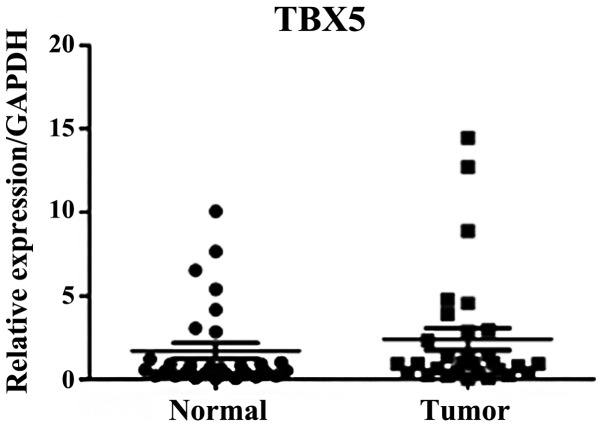
TBX5 mRNA expression in stage III and IV gastric cancer tumor tissues (n=30) was detected by reverse transcription-quantitative polymerase chain reaction in tumor vs. adjacent non-tumor tissues (P=0.01, Wilcoxon matched-pairs signed-rank test). TBX5, T-box transcription factor 5.

RT-qPCR was performed on 60 pairs of surgical specimens (tumor and adjacent non-tumor tissue samples) to examine the mRNA expression levels of TBX5. A significant difference was identified between the stage I and II tumor and paired non-tumor tissue samples (P=0.01; Fig. 1). However, no significant difference was observed in TBX5 mRNA expression levels in the stage III and IV GC samples compared with the adjacent normal tissues (P=0.318; Fig. 2).

#### Immunohistochemical analysis and clinicopathological characteristics

**Figure 3.**
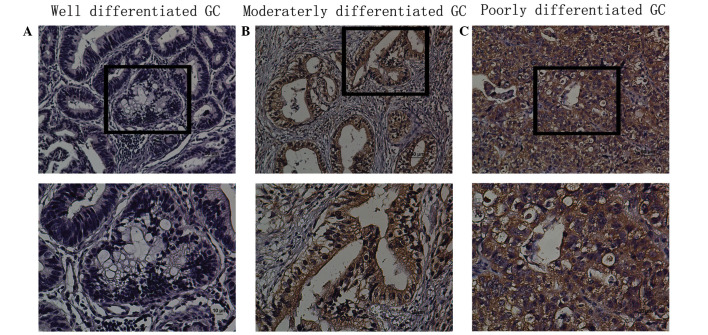
The *in situ* expression of TBX5 protein in stage I and II GC specimens was assessed by immunohistochemistry. (A) Negative TBX5 staining was observed in well-differentiated GC. (B) Weak TBX5 staining in moderately differentiated GC. (C) Strong TBX5 staining in poorly differentiated GC. (Upper panels, magnification ×200; lower panels, magnification ×400). TBX5, T-box transcription factor 5; GC, gastric cancer.

**Figure 4.**
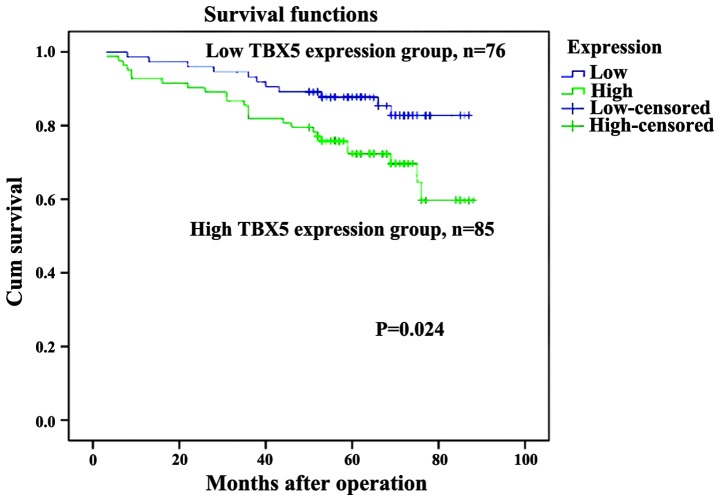
Kaplan-Meier survival analysis of patients with stage I and II gastric cancer (n=161). The survival rate in the high expression group was lower than that of the low expression group (log-rank test, P=0.024). TBX5, T-box transcription factor 5.

The protein expression levels of TBX5 *in situ* were evaluated by immunohistochemical analysis of paraffin-embedded GC tissue blocks (n=161). TBX5 was expressed in a nuclear and cytoplasmic pattern in tissues, and TBX5 protein expression was observed in the tumor tissue (Fig. 3). The expression of TBX5 was high in poor-differentiated group and low in well-differentiated group. TBX5 expression was ‘low’ in 76/161 (47.2%) and ‘high’ in 85/161 (52.8%) as assessed using the criteria mentioned above. No correlations between the clinicopathological variables and TBX5 expression were observed (Table I). As demonstrated in the Kaplan-Meier survival curves, TBX5 expression may be used to predict the OS of stage I and II GC (P=0.024, Fig. 4). The expression of TBX5 was demonstrated to be a significant prognostic factor for patients with GC following univariate analysis (P=0.028; Table II). In addition, TBX5 expression was identified as an independent prognostic factor in the multivariate Cox proportional hazards model analysis (P=0.017; Table II).

## Discussion

The aim of the present study was to observe the expression of TBX5 in primary GC samples, in addition to identifying its potential clinical relevance.

T-box (TBX) transcription factors belong to a conserved gene family with critical roles in organogenesis and embryogenesis (11). TBX5 is a member of the T-box family and is essential for the embryonic development of the forelimbs and heart (4,5). HOS is caused by mutations in TBX5 (12). In a previous study by Rosenbluh *et al* (9), it was demonstrated that TBX5 forms a complex with β-catenin and YAP1, which is essential for the process of tumorigenesis in colorectal cancer. Numerous previous studies have reported that β-catenin may be associated with GC (13,14). Therefore, additional studies are required to investigate the potential association between TBX5 expression and clinicopathological features and survival data in GC. The present study evaluated the expression of TBX5 in GC patients who received uniform treatment and determined its clinicopathological significance by correlating this data with the characteristics of the patients and long-term follow-up information. The findings of the present study indicated that TBX5 may be a useful biomarker to identify patients with stage I and II GC who may have unfavorable survival rates.

In the present study, RT-qPCR analysis was used to determine that the mRNA level of TBX5 was reduced in normal paracancerous tissues compared with stage I and II GC tumor tissues (P<0.01). However, no significant difference was demonstrated in TBX5 mRNA expression levels in tissue samples from patients with stage III and IV GC compared with normal tissues. These results indicated that TBX5 expression may be involved in the progression of stage I and II GC. Immunohistochemical analysis demonstrated that high expression of TBX5 was detected in 52.8% (n=161) of the GCs. The clinical and pathological significance of TBX5 expression in GC was systematically evaluated; however, no significant correlation was observed between disease characteristics and the level of TBX5 expression. Since the present study was a single institute retrospective analysis, further studies are required to evaluate the potential association between TBX5 expression and clinicopathological features in other populations.

Kaplan-Meier survival analysis demonstrated that the TBX5 expression level was a significant and independent predictive factor in cases of surgically resected stage I and II GC. High TBX5 expression was observed in patients with significantly shorter median OS, compared with patients with low expression of TBX5. Rosenbluh *et al* (9) demonstrated that the YAP1/β-catenin/TBX5 complex is localized to the Bcl-2-like protein 1 and baculoviral IAP repeat containing 5 promoters (15). This is in accordance with another previous study that demonstrated that TBX5 forms a complex and induces transcription of atrial natriuretic factor (16). The transcriptional factors were observed to regulate developmental and cancer-associated phenotypes (17). Rosenbluh *et al* (9) also demonstrated that TBX5 was a key transcription factor target of the β-catenin/YAP1 complex, which regulated cancer phenotypes. Therefore, TBX5 may be activated and overexpressed in stage I and II GC. The mechanisms underlying the potential function of TBX5 were explained in the study by Rosenbluh *et al* (9). Wnt/β-catenin signaling has been demonstrated to be involved in the pathogenesis of cancer and is essential for cancer initiation and progression (18). YAP1, β-catenin and the transcription factor TBX5 form a complex and move to the promoters of anti-apoptotic genes, including BCL2L1 and BIRC5 through the phosphorylation of YAP1 (9). This hypothesis has been investigated in cell lines and animal models (4–6,8). Collectively, these data demonstrate that TBX5 may be a novel biomarker that is potentially an independent predictor of the survival rate of patients with stage I and II GC and that high expression of TBX5 may aid in distinguishing which patients with stage I and II GC may have unfavorable survival rates.

At present, TNM stage is widely accepted as a powerful predictive parameter of survival rates (19). However, cases of the same TNM stage are often observed to result in varied clinical outcomes, and TNM alone may not be sufficient to predict clinical outcomes. Therefore, it may be useful to determine those biomarkers that may aid in the identification of patients with potentially poor survival rates within the same TNM stage, so that they may be selected for specific treatments. Having demonstrated the clinicopathological significance of TBX5 expression in the prognosis of OS in patients with GC, additional studies are required to investigate the significance of TBX5 in patients with stage I and II GC treated with chemotherapy, and the association between TBX5 and β-catenin.

In conclusion, patients with stage I and II GC and high expression of TBX5 resulted in unfavorable survival rates compared with those with low expression of TBX5. The present study demonstrates that the expression level of TBX5 in stage I and II GC following surgery may be a potential prognostic biomarker of survival rates in patients with GC.

## Figures and Tables

**Table I.** The expression of TBX5 and the clinicopathologic characteristics of patients with gastric cancer stage I and II.

Characteristics (n)	Low TBX5 expression (n)	High TBX5 expression (n)	χ^2^	P-value
Gender			0.117	0.740
Male (108)	52	56		
Female (53)	24	29		
Location			1.565	0.687
Fundus of stomach (68)	31	37		
Proximal (25)	14	11		
Distant (65)	29	36		
Total (3)	2	1		
Tumor invasion (T)			0.071	0.968
T1 (33)	15	18		
T2 (31)	15	16		
T3 (46)	21	25		
T4a (51)	25	26		
Nodal status (N)			2.028	0.363
N0 (124)	55	69		
N1 (31)	17	14		
N2 (6)	4	2		
TNM staging, 7^th^ed.			0.15	0.698
Stage I (49)	22	27		
Stage II (112)	54	58		

TNM, tumor/node/metastasis; TBX5, T-box transcription factor 5.

**Table II.** Univariate and multivariate analyses of overall survival in 161 patients with stage I and II gastric cancer.

	Univariate analysis			Multivariate analysis		
		
Variables	HR	95% CI	P-value	HR	95% CI	P-value
Age	1.016	0.986–1.046	0.295			
Gender^a^	1.363	0.697–2.666	0.365			
Location^b^	0.57	0.385–0.846	0.005^e^	0.65	0.435–0.97	0.035^e^
TNM^c^	5.623	1.724–18.339	0.004^e^	4.699	1.417–15.585	0.011^e^
TBX5^d^	2.213	1.088–4.501	0.028^e^	2.378	1.168–4.844	0.017^e^

a Male vs. female

b fundus of the stomach/proximal/distant/total

c staging I/II

d high vs. low

e P<0.05. CI, confidence interval; HR, hazard ratio.
